# Patients’ experiences and perceptions on associates of TB treatment adherence: a qualitative study on DOTS service in public health centers in Addis Ababa, Ethiopia

**DOI:** 10.1186/s12889-018-5404-y

**Published:** 2018-04-10

**Authors:** Zekariyas Sahile, Abenezer Yared, Mirgissa Kaba

**Affiliations:** 1 Department of Public Health, College of Medicine and Health Sciences, Ambo University, P. O. Box 19, Ambo, Oromia Ethiopia; 2 Department of Sociology, College of Social Sciences and Humanities, Ambo University, P. O. Box 19, Ambo, Oromia Ethiopia; 3 School of Public Health, College of Health Sciences, Addis Ababa University, P. O. Box 11950, Addis Ababa, Ethiopia

**Keywords:** Tuberculosis, DOTS, Adherence, Phenomenology, Ethiopia

## Abstract

**Background:**

Ethiopia is one of the countries with the highest TB burdens in the world. There are multitude of challenges related to the implementation of DOTS and adherence to treatment. This study aimed to assess patients’ experiences and perceptions on associates of TB treatment adherence in Addis Ababa, Ethiopia.

**Methods:**

A phenomenological approach was employed to generate qualitative data through the in-depth interview of TB patients attending DOTS in two public health centers. A total of ten participants, who were purposively selected till conceptual saturation was reached, were interviewed using topic guides prepared in line with the study objectives. Interviews were tape-recorded, transcribed verbatim, and translated to English. Open Code software was used to facilitate analysis. Themes pertaining to patient, health service, therapeutic, and socioeconomic factors were developed, and findings were presented accordingly.

**Results:**

Experience of missing medications was reported by a single participant. Most informants pointed out that TB is transmitted through coughing and expectorate, and prevented by letting in open air in public gatherings. However, most of them stated cold air and few mentioned contaminated food as causes of TB. Perceived risk of non-adherence to medication was among recounted reasons behind treatment adherence. Some informants also recalled to have had the intention of withdrawing medication due to perceived wellness, which they actually did not change into action. Most of the participants generally had smooth relationships with their DOTS service providers. Even if more than half of the patients preferred follow-ups by the same professional, most received DOTS service by two or more service providers.

**Conclusions:**

TB treatment non-adherence was not found to be a major challenge among the study participants. Perceived risk and wellness were implied to be responsible factors for adherence. Albeit the fact that few informants encountered unethical behaviors by some health professionals, interviewed patients generally had positive evaluation of the patient-provider relationship and the DOTS service obtained. There is a need to train and monitor DOTS service providers and ensure the provision of DOTS service by the same provider throughout the treatment period of a given patient.

## Background

Tuberculosis (TB) remains a major global public health problem and continues to be responsible for ill-health among millions of people each year. In 2015, TB was one of the top ten causes of death worldwide and was ranked above HIV/AIDS as one of the leading causes of death from infectious diseases [1]. In Ethiopia, the estimated cases of TB in 2013 were 210,000 with incidence rate of 224 cases per 100,000 people [2]. Indeed, compared to the baseline magnitude documented in 1990 [3], the national TB prevalence and mortality rates due to TB respectively decreased by 50.5 and 64% in 2013. However, Ethiopia is classified as one of the worst affected 30 high burden countries for TB in the world [4], as TB continues to be the major health problem and one of the leading causes of mortality from communicable diseases.

Since 1994 the World Health Organization (WHO) has launched the Directly Observed Treatment, Short Course (DOTS), a brand name for the internationally recommended strategy for TB control. The DOTS strategy warrants to identify infectious TB patients and cure using standardized drug combination. A standardized TB prevention and control program incorporating DOTS in Ethiopia began in 1992 as a pilot in Arsi and Bale zones of Oromia regional state and later expanded to other areas of the country [5].

Despite the improvements in TB treatment outcomes evidenced by some studies since then [6, 7], further researches indicated that lots of problems were observed in the course of administering DOTS services in the country. For instance, indicating TB patients on DOTS had to overcome many challenges to comply with TB treatments on daily basis, studies conducted in the capital Addis Ababa pointed out its undesired implication on work and social lives as emerging problems of TB patients. Coupled with lack of or high cost of transportation, daily treatment was especially very challenging and physically demanding for severely ill patients [8, 9]. Apart from the implication it had on patients, health care professionals also pointed to difficulties in implementing facility-based DOTS as per the recommended full course [8, 9].

Patient compliance is a key factor for the success of any treatment as quality health care outcomes depend on patient’s adherence to recommended treatment regimens. Non-compliance with TB treatment poses a significant public health threat, as it is associated with increases in transmission rates, morbidity, and costs to TB control programs [10]. Moreover, non-compliance leads to persistence and resurgence of TB and is regarded as the chief cause of relapse and drug resistance [11]. However, significant proportion of TB patients in many countries stop treatment before completion [12]. In Ethiopia too, considerable number of patients non-adhere to TB treatments at different stages. A study conducted in Addis Ababa showed that 19.6% of patients were non-adherent at the early stage of TB treatment and 25% were non-adherent at the end of the treatment [13]. Another study in the same study area indicated that 3.7% of TB patients died during follow-up, 5.1% were reported as defaulters, and 0.4% were documented as treatment failure [14].

Although interplay of factors determine TB treatment adherence and outcomes, and adherence itself is a concept that allows for comprehensive assessment of correlates of medication intake such as characteristics of the regimen, attitudes of service providers as well as socioeconomic, cultural and environmental factors [15, 16], many studies merely addressed the knowledge, attitude and beliefs about TB treatment. Some of these researches centered their analyses on the influence of patients’ understanding about duration of treatment and consequences of defaulting as determinants of adherence to treatment [10, 17]. Focusing exclusively on such patient-related personal factors, therefore, these studies failed to accord due emphasis to other contributing factors, including socioeconomic and health service-related associates, that also influenced patients’ decision to either adhere or non-adhere to required medication follow-ups.

Holistically exploring TB patients’ experiences and perceptions on associates of adherence to medications, as attempted in this study, is thus crucial for designing proper and alternative interventions through patient-centered approach by concerned bodies. In addition, asking patients about the services they obtained is considered as a vital quality indicator of health service. A study with such approach, by providing information on facilitators and barriers to adherence, produces essential inputs to improve treatment outcomes as well as to reduce the spread of drug-resistant TB. Bearing in mind the need for continual assessment of DOTS implementation and gaps identified in this regard, therefore, this study aimed to assess patients’ experiences and perceptions on associates of TB treatment adherence in public health centers providing DOTS service in Addis Ababa, Ethiopia.

## Methods

### Study design and setting

With the aim of exploring the experiences and perceptions of TB patients on associates of treatment adherence, phenomenological approach was employed to carry out qualitative data collection [18]. Accordingly, in-depth interviews with TB patients were conducted from 19th to 25th of August 2016 in two public health centers found in ‘Nefas Silk Lafto’ sub-city. With the total population of 396,486 [19], ‘Nifas Silk Lafto’ is one of the ten sub-cities in Addis Ababa, Ethiopia.

### Study participants and sampling technique

Purposive sampling technique was used to identify TB patients following DOTS service in the two public health facilities. Age (18 years old and above) and duration of treatment (3 weeks or more) were considered for criteria for selection. Nurses at the TB clinics assisted in the screening and selection of the study participants when they came to the health facilities for regular DOTS in the morning. Selection continued until conceptual saturation was reached (to the point no further new information was obtained any more), and a total of ten TB patients on DOTS (five in each facility) were interviewed.

### Data collection methods and procedures

Literatures were reviewed to develop an interview guide which was first prepared in English and later translated to Amharic (the local work language). Data were collected by the first author who took intensive trainings on qualitative research and data collection. Each depth interview session lasted for about an average of 45 min. All interviews were recorded using a digital audio recorder.

### Data analysis methods

Verbatim transcription and translation to English were carried out. Consistency of transcripts were checked by two independent readers, who were also asked to help identify themes based on objectives of the study. The team members discussed and agreed on the final themes analyzed. Accordingly, four major themes (patient, health service, therapeutic and socioeconomic) and specific sub-themes under each theme were developed (Table 1). Open Code software version 3.5 was used to facilitate data analysis. Responses were categorized under each theme and sub-theme. Interpretations of the qualitative data were dependent upon patients’ descriptions of their experiences and perceptions, which the researchers checked against the verbatim transcripts for accuracy and consistency.

**Table 1:** Themes and codes on TB treatment adherence associates identified from TB patients’ in-depth interview in Addis Ababa, August 2016 (*n = 10*)

Themes	Codes
Patient-related	Beliefs on TB curability, causation and transmission, and prevention
	Substance use
	Forgetfulness
	Perceived risk of non-adherence to medication
	Perceived wellness
Health service-related	Accessibility
	Waiting time
	Patient-provider interaction
	Patient’s preference
	Health care evaluation
Therapeutic	Side effect of medication
Socioeconomic	Food accessibility and consumption
	Social support
	Discrimination

### Ethical considerations

Ethical clearance was obtained from the ethical review committee of the School of Public Health, Addis Ababa University. In order to secure privacy of the informants, the in-depth interviews were conducted in a separate room after the selected patients completed their treatments at the TB clinics. All interviewees were informed about the objectives, data collection procedures, possible risks and benefits of taking part in the research, and confidentiality of the obtained information. The informants then voluntarily decided to participate, and oral consent was obtained from each of them.

## Results

### Background profile

Out of the total of 10 TB patients who took part in the study, seven were males and coincidentally, all were single. Seven were below 30 years old and the rest were 30 to 40 years of age. While six participants attained secondary education, eight were employed (3 self- and 5 private organization employees). Six of the interviewed patients had extra pulmonary TB and the remaining four had pulmonary TB (Table 2).

**Table 2:** Background profile of TB patient study participants in Addis Ababa, August 2016 (*n* = 10)

Characteristics		Participant									
		A	B	C	D	E	F	G	H	I	J
Sex											
Male	7	M	M		M	M			M	M	M
Female	3			F			F	F			
Age group											
18–23	3	18	19	19							
24–29	4				24	25	26	28			
30–35	2								33	35	
36–41	1										38
Marital Status											
Single	10	S	S	S	S	S	S	S	S	S	S
Married	0										
Educational level											
Primary, grade 1 to 8	1					4					
Secondary, grade 9 to 10	6	9	10	9			9	10	10		
Preparatory, grade 11 to 12	1									12	
Higher, 12+	2				12+						12+
Employment status											
Student	2	S		S							
Self-employee	3					SE	SE				SE
Private organization employee	5		POE		POE				POE	POE	
TB category											
Pulmonary TB (PTB)	4	PTB							MDR-PTB	PTB	PTB
Extra pulmonary TB (EPTB)	6		EPTB	EPTB	EPTB	EPTB	EPTB	EPTB			

### Adherence to TB treatment

One among the total of ten participants had the experience of missing medications. Including this participant, few other patients also admitted taking their medications at the wrong time (not on time). Revealing her experience of instances of treatment non-adherence, a 19 years old extra pulmonary TB patient further disclosed that she did not miss her medications “for consecutive days, but missed medications for five days.”

### Experiences and perceptions on associates of TB treatment adherence

Findings pertaining to each of the major themes identified from the analysis of qualitative data on TB patients’ experiences and perceptions on factors related to TB treatment adherence (Table 1) are presented below with thick descriptions and demonstrative quotes.

### Patient-related factors

#### Beliefs and perceptions on TB curability and treatment, causation and transmission, and prevention

##### Beliefs and perceptions on curability and treatment of TB

In line with almost all of the interviewees’ beliefs and perceptions on curability and treatment of TB, a 33 years old male MDR TB patient, asserting that TB is definitely curable, stressed that “MDR TB, however, is very dangerous if the patient does not follow the medication appropriately, and failure to do so not only harms the patient but also infects others.”

Another pulmonary TB patient of the same sex and approximately similar age added, “I am responsible for the cure… Since I started medication, I quitted all the substances that I used to abuse. If I keep on following the treatment as such till I complete the six months, I will be cured.” However, one female patient aged 19 was skeptical about the curability of TB and effectiveness of the treatment itself. She said, “I do not know… It (*the TB treatment*) only subsided the swelling on my neck. I still have a headache and only one side of the swelling subsided. There is pain, but it is fine/moderate. I think it (*the TB disease*) may be cured after two months, but it is not gone until now.”

##### Beliefs and perceptions on causation and transmission of TB

Most participants had lay beliefs on TB causation. Majority of the interviewees believed that TB is caused by cold air and few participants reported it is caused by virus and contaminated food. A female extra pulmonary TB patient aged 19 years stated “cold air… like during rain” caused TB and shared her experience by reporting, “I used to share a bed with my grandmother who had TB…Asthma. And people used to ask me why I sleep with a person who had such coughing. But, I got TB not through transmission from another person. I think my TB was caused by cold air.” A male pulmonary TB patient of similar age added that “the cause of TB is contaminated food, for example, eating cold meal from refrigerator and consuming contaminated food.” Concerning TB transmission, however, unlike misconception about its cause, most informants explained that TB is transmitted through exposure to coughing, expectorate, and breathe from TB patient.

##### Beliefs and perceptions on TB prevention

Most participants mentioned covering the mouth when coughing, putting expectorate in distance, opening windows, and avoiding close contact with infected patient as methods of preventing TB transmission. Furthermore, they were aware that extra pulmonary TB is not transmittable. A 38 years old male with pulmonary TB told that “opening windows in public places is necessary as no one knows who is healthy or not” to prevent TB transmission. A 24 years old male with extra pulmonary TB explained how he was infected and how transmission could be prevented by stating:

“For about a year, I have been closely working with a person who continuously coughed for the whole year. At the end of the day, the bacterium was transmitted to me and I also developed TB. I had small swelling around my neck. Had that person been treated early, he would not have transmitted the disease to me. Whenever anyone experiences dry coughing, he/she needs to get early treatment before transmitting to others. Opening windows in public areas is also helpful to prevent TB transmission.”

However, two participants, who also had misconceptions regarding TB causation, had unscientific beliefs about TB prevention. These were a 19 years old female extra pulmonary TB patient and an 18 years old male pulmonary TB patient who respectively said that “only Allah can prevent it” and “TB can be prevented by taking foods with vitamins.”

#### Substance use

Participants specified that practices like chewing chat/khat, drinking alcohol and smoking cigarettes negatively affects the therapeutic course, and mentioned that cigarette affects the lungs as TB does, khat decrease appetite, and alcohol affects the liver. A 33 years old male on MDR TB treatment, explained his experience of smoking cigarettes, chewing khat, and drinking alcohol, said “I have now stopped using all these substances once and for all; I was ill in 2005 and now again, and I do not want to come again”, and recommended that “preventing oneself from use of substances is very important.” Another 35 years old male added that “I, for example, started having good food and quitted all addictions like khat, cigarette, alcohol, and sex; alcohol is not used with any type of medication in the first place, and cigarette smoking exposes to lung and respiratory problems, similar to TB.”

#### Forgetfulness

For patients with the experience of not taking their medication on time and missing doses of medications, forgetfulness was one of the reasons for interruptions. Furthermore, patients also forgot their medications because the daily DOTS visit was not fully implemented. A 19 years old female reported, “I sometimes forget to take my medications… I also forget to take it on time… For instance, I took the medication at 10:00 am while I should have done it early in the morning.” The same participant also reported missing doses of medications due to forgetfulness. A 24 years old male respondent added, “I took all my medications. But, I once I forgot the time and took the medication hours after regular time.”

##### Perceived risk of non-adherence to medication

Representing the commonly mentioned perceived risks of non-adherence to medication, an MDR TB patient said, “one may not feel it now but discontinuing treatment entails risks; when one gets minor infections like flu or pneumonia, it may facilitate relapse of TB, and once the disease is transferred to MDR TB it is becomes difficult to treat.” By reporting what other patients encountered due to non-adherence, another participant perceived risks as, “discontinuing medication may lead to TB that is difficult to cure and requires long term treatment. I have seen a patient in this facility. Instead of two months, he is undergoing injections for eight months now and this is because he discontinued his medication.”

##### Perceived wellness

Feeling of wellness was one of the reasons reported by patients for their intention to discontinue their treatments. Two participants reported they intended to discontinue their medication because they were feeling well. However, they eventually changed their mind and decided to continue medication after having discussion with health care providers on adverse consequences. A male 19 years old participant reported, “I told the nurse that I have recovered from my pain and asked her why I should continue taking the medication for the next months.... But, she advised me to complete my treatment and warned me the disease may relapse if I discontinue.” Another 38 years old informant of the same sex similarly said:

“When they (*health professionals*) told me to continue medication for the next four months, I disagreed. I said no; I am cured and the test result for the past two months was negative. They then explained that doing so will have side effects and the treatment should be followed till I complete six months. As result, I decided not to quit the medication.”

#### Health service-related factors

##### Accessibility

The health facilities were located close to majority of the interviewed patients’ residences and it only took them 5 to 45 min of walking to reach the respective centers. Two participants, however, said the health facilities were far from their homes and relatively costly for transportation services. In spite of the distance, they additionally told it posed no difficulty to their TB treatment follow-ups even during the first 2 months of daily DOTS visits.

A 28 years old female extra pulmonary TB patient responded, “I pay two Ethiopian Birr to come to this facility. Since I have to go back to my workplace, I should pay twice for transportation. Had I been able to take the medication at home, I could have gone to my work directly. But it is okay.” Another 25 years old male extra pulmonary TB patient added, “my home is too far from this facility… It costs me ten Ethiopian Birr for transportation. Of course, there is another health facility around my residence, but it is not convenient with my job as my workplace is around this health center. Thus, I do not mind following my treatment here.”

##### Waiting time

The Patients generally reported they were able to meet the health professionals at the centers in few minutes (maximum of 5 min). Most of them said waiting time was getting lesser and lesser after the first 2 months of treatment. A male patient of 24 years of age, agreeing with the majority that waiting time was longer during the intensive treatment phase (the first 2 months), “at the time I started medication, there was a crowd and long queue. But now, because I come here once a week and take my medication at home, I come late after other patients went so that I can avoid waiting for long.” Few participants experienced longer waiting times because the health professionals come late to the health facilities and because different health professionals worked at different times in the TB clinics. In this regard, a 28 years old female extra pulmonary TB patient from a different health facility disclosed that the service provider professionals “rotated at different centers and I met different health professionals at different times; and they were not sometimes available, and they came when called (*through phone*). In one occasion, I waited for about three hours.”

##### Patient-provider interaction

Most patients reported having good relationships with health care service providers at the TB clinics. Participants further said they benefited from the information they obtained from professionals on duration and side effects of treatment, risks of non-adherence as well as dietary practices to follow and habits to avoid. A male pulmonary TB patient shared:

“Beyond being a very good person, my first provider was very professional and committed. She helped me a lot; like a mother, she encouraged me to stay strong. ‘How much time are you left with? Only a few!’ she usually said to me. All these were despite the fact I sometimes showed irritable behavior due to my unfavorable experiences and occasionally tended to raise conflicts. When I complete my treatment, I want to write an appreciation letter to her and put it in the comment box.”

From a different health facility, a female patient, 26, added, “She (*the health professional*) understood me well whenever I told her about my illness experiences. She has been a very good person to me. She was keen in listening and understanding me.” However, a pulmonary TB patient, a 38 years old male, who had the experience of taking his injections on weekends (days off) by other professionals from other wards reported unwelcoming encounters with the health care providers. He described:

“On Saturdays and Sundays, I took my injection in another room since my usual service provider was on days off on weekends. When some of these professionals saw me, they immediately wore mask… as if they were in a battle or war! … A given service provider, for example, opened the door as I came in to the room and impolitely ordered me to turn my face against her. Such behavior is so unethical. I do not know… It may seem as if I am philosophizing but I believe pain is psychological; and added to TB, it makes the time for recovery longer. One of the days, I argued with her that she should not be acting as such… my disease is not Ebola! She finally also admitted that she has been exaggerating.”

An MDR TB patient in the same health facility similarly noted that:

“One of the issues in this facility has to do with service providers in the reception room; they are disrespectful to TB patients... Once, the health professional was late to give us our medications and the time already passed. When we told the receptionist about the issue, she unpleasantly and disrespectfully replied that she does not care at all and that it is not her business whether or not we are TB patients… Such disgusting behavior and reaction is not accepted and should not be repeated.”

##### Patient preferences

Although six of the study participants preferred treatment follow-ups provided by a single professional, most of the participants, except two, reported receiving DOTS service by two or more health care providers. A 28 years old female participant said, “I met different professionals at different times. The present one is my fourth medication provider. It is good if treatment is provided by one professional because new professionals inquired about my status and information over again.” Frequent change of providers also resulted in treatment inconsistencies, regarding period of completing medication for example, the above participants additionally revealed that “the previous health professional said I was scheduled to complete my medication on the 9^th^ of September. The current provider, however, told me the final date of my treatment will be on the 7^th^ of October 2016.” Another patient, 19, of the same sex and health center who was similarly following her medication by a fourth professional stated her preference and explained her reasons as follows:

“I prefer if follow-up would be performed by a single professional throughout the treatment period, because that one doctor thoroughly knows about my disease and behavior. I prefer to be treated by the one that I know well and share my information without any fear. For example, at times of weight loss, new professionals did not notice the change and compare it with the previous weight of the patient.”

Reporting that he got about 90 % of his follow-up of first 2 months’ treatment from one and the same service provider before having another professional later, a participant from a different health facility responded that he would prefer to be served by a single health professional but stated he would tolerate if this was not the case too by explaining:

“I would rather prefer to be treated by a single professional throughout the medication period. For example, I was so familiar with my first nurse. But it is difficult to say this woman (*the nurse*) should always work here (*TB clinic*). While I was discussing with this nurse, she told me that working here for six months and more is risky as there are different types of TB and as some patients refuse to be cautious and wear a mask. As a result, no health professional preferred and worked here for more than six months. If it were possible for the professionals to stay and work longer, I would prefer to be served by only one service provider. When the professional called me by my name, warmly greeted and welcomed me, and freely discussed with me, it meant a lot to me. Nonetheless, due to the risks on the providers, I would tolerate changes and replacements of the professionals.”

##### Health care evaluation

The participants generally evaluated the DOTS services they received as very good. Accordingly, while a 33 years old male said that “the service is very good for I got my injections as soon as I came in the morning by nice and respectful health professionals who came early, and this is especially good for MDR TB patients and has improved from previous times”, a participant of the same sex aged 24 years added “the very good thing is the fact that the treatment is provided for free and the services are available in nearby and easily accessible health facilities.” Another participant of 38 years old explained that “the treatment I have been receiving helped me a lot to quickly recover from my illness. I think the service provided is satisfactory in the context of Ethiopia as a developing country. But if it is possible to avail so much more, who would hate that?”

The participants further suggested areas for future improvement. A 24 years old male participant commented that “there is indeed a need to increase the number of service providers and TB treatment rooms in order to address the problem of patient overcrowdings and probably overload of cases on the part of the professionals.” An interviewee, a 33 years old male, added:

“Services provided by professionals from other departments should be improved. The health center administrator should device a mechanism to monitor these people. They should respect patients; they did not even ask what you want or need. This should be corrected by having meetings and discussions with them, or there should be other means to regularly evaluate these staff members.”

This participant also reported that he was obliged to purchase distal water for injection from private clinics because it was sometimes unavailable at the public health centers. Another participant, a 35 years old male, recommended the health professionals to refer difficult cases to senior doctors by explaining his experience as:

“I had cough until I completed the first two months of medication. I do not know whether it was due to my carelessness or exposure to cold air. I asked them (*the professionals*) and they told me to wait for few days. I waited but no there was no improvement. I then requested them to refer my case to other senior physicians but they refused to do so. Left with no other options at this public health center, I finally went to a private clinic to find out the cough was not related to TB. I got treatment there and became well.”

#### Therapeutic factors

##### Side effects

Most participants reported experiencing TB medication side effects and commonly mentioned having red urine, headache, nausea, diarrhea, stomach upset, vomiting, rash, itching, and change in the color of the eyes. Only few told they did not experience any side effect. However, none of the participants missed their medications or had the intension of discontinuing their treatments due to these medication side effects. Most of the participants further said the side effects disappeared after their adaptation to the medication. A 28 years old patient shared her experience by stating, “at the beginning, the medication was not comfortable to my body... I had rash and itching. I was told to wait until the drug adapts to my body. And as I get used to the medication later on, the side effects disappeared.”

#### Socioeconomic factors

##### Food accessibility and consumption

Almost all the patients were able to get food whenever they needed to, and that the medication increased their appetite for food, except one participant. A 28 years old female reported “since I spend much of my time at my workplace, I purchase and eat whenever I need food. If I do not have money, I can borrow from my colleagues and get food.” A patient aged 26 years added, “though I do not have any problem in food access, my appetite has decreased. I eat the available food, but I do not drink milk and consume avocado instead. The professionals told me to drink milk but I dislike milk and I never drink milk, but I eat meat.”

However, a 19 years old male participant responded that he was not able to get adequate food when he needed. Even though his appetite increased, he could not afford buying the food items like milk and egg recommended by health professionals and lost weight as a result. He unveiled, “I could not eat what they (*the health professionals*) told me I should eat. I do not have the money to afford that. My weight decreased. Since I started the medication my appetite has increased but I could not get enough food.”

##### Social support

Almost all study participants reported having the required support from their respective family members. For example, while an 18 years old male said, “I get support from my family. They prepared different kinds of meals for me including egg and meat”, a 28 years old female added, “my brothers remind me to take my medication and they help me get milk and egg as far as possible.” However, a male teenager reported not having any kind of support from anybody. He claimed, “I do not get any support from anybody… My family does not reside here (*Addis Ababa*).”

#### Discrimination

Most participants, except two, experienced no discrimination from their family and community due to the fact that they had TB. According to this majority, they were not discriminated partly because they had extra pulmonary TB which is not transmittable. An extra pulmonary TB patient, a female aged 26, stated, “It is me who used to discriminate others, people rather supported me. I used to discriminate them until I knew my TB is not transmittable, it is intestinal. Why I used to do this was because there were children at home.”

A TB patient in her late teen added, “… I asked the health professionals whether the disease is transmittable and I should separate my food plate. Indicating that I did not have cough and sputum, they told me the disease affects only me… And I never experienced any form of discrimination from anybody.” However, an interviewee, 35 years old male, reported experiencing discrimination from his family because workers in the reception and medical record room gave exaggerated information regarding TB transmission to members of his family. He shared his experience as follows:

“The information on carefulness given by workers in the medical record and reception room was so exaggerated. They misinformed my family members that they should never close the doors and windows of the separate room I was made to live in alone. All the doors and windows were always open, be it during the day or night, which exposed me to cold air and thus additional disease. Even relatives, friends, colleagues and neighbors who came to ask me whether the disease is transmitted after I start the medication and I answered them I do not know.”

A 38 years old male pulmonary TB patient, referring to the lack of awareness and non-scientific personalistic understanding of illness causation that prevailed among members of his community, told that “some people considered TB as an evil, a curse, a punishment from God. Of course, TB is transmittable disease and I now know that there are different types of TB; but it does not just bite you when your immunity is well. Is not it? I just cannot get the idea why people exaggerated it so much.”

## Discussion

Among the total of ten TB patients who participated in the study, one reported missing medications and few patients disclosed missing the exact time of taking medications as recommended by health care professionals. Identified from the analysis of the qualitative data, findings of the study came up with four major themes on TB patients’ experiences and perceptions regarding factors related to DOTS adherence: patient-related, health service-related, therapeutic, and socioeconomic factors.

Beliefs and perceptions on curability and treatment, causes and transmission, and prevention of TB; substance use; forgetfulness; perceived risks of non-adherence to medication; and perceived wellness were the sub-themes categorized under patient-related factors. Almost all participants believed that TB is curable if medication is taken properly and followed for 6 months, and if use of substances/drugs is avoided. Seen in relation to adherence, belief in the curability of TB was similarly mentioned as a factor that positively influenced adherence to treatment in another study [20]. As opposed to these participants’ knowledge on duration of TB treatment, other studies carried out in Asia reported that the long treatment period was poorly understood by patients [10, 21].

Consistent with finding of a study conducted in Addis Ababa [20], most participants, however, had misconceptions regarding the cause of TB, for they reported cold air and contaminated food as causal factors. Contrary to their misconceptions and lay beliefs on TB causation, most informants were well aware of its transmission and prevention as they correctly pointed out that TB is transmitted through coughing, expectorate, and breathe from TB patient, and prevented by covering mouth when coughing, putting expectorate in distance, and opening windows in public places. They were also aware that extra pulmonary TB is not transmittable.

For patients who reported to have the experience of not taking their medication on time and missing doses of medications, forgetfulness was one of the reasons behind such interruptions. In line with reports indicating adherence appeared to be facilitated where patients understood the importance of fully completing treatment [10, 17], participants of this study were aware of this requirement to restore their health and commonly mentioned perceived risks of non-adherence to medication including relapse of the disease and cause of MDR TB which is difficult to treat and could lead to death.

Perceived wellness was also one of the reasons reported by some patients for the intention they had to discontinue their treatments. Correspondingly, patients in India and Pakistan stopped treatment because they felt better and perceived they were cured [17, 21]. While a case report in Malaysia also indicated that false perception of being cured was a reason for non-adherence [22], a study in Thailand reported perception of health status was statistically associated with adherence to treatment [23].

Health service-related factors (accessibility, waiting time, patient-provider interaction, patient’s preference, and health care evaluation) indicated that health facilities were located close to patients’ residences, supporting the view that patients could regularly attend treatments if their home were close to a clinic. But, even for the few participants who reported their homes were far away from the DOTS provision centers and costly for transportation services, the issue of accessibility in terms of physical distance posed no difficulties and inconveniences to their TB treatment follow-ups.

This finding was against a qualitative study that indicated daily visits to health facilities for DOTS in Addis Ababa was difficult because of distance from patients’ residence, lack or high cost of transportation and undesired implications on their work and social lives [9]. These differences could be explained in terms of two interrelated factors. First, it might be due to the expansion of health facilities that provided DOTS service in the city, which in turn might have improved access to treatment. Secondly, since daily DOTS visits was implemented only for the first 2 months, this might have resulted in the favorable experience of following TB treatments without difficulties by participants of the present study.

As to the finding of this qualitative research, patients generally reported they were able to meet the health care professionals at the centers only after waiting for few minutes (maximum of 5 min), and only few of them said they sometimes experienced longer waiting times because the professionals came late to the health facilities. Studies identified that problems manifested at health facilities included long waiting times, queues, and inconvenient appointment times [10, 21, 24], and reported that patients experienced difficulty in accessing treatments because of inconvenient opening hours of centers and provider absenteeism [21, 25].

Concomitant to their reported interaction dominated by mutual understanding and respect, the patients generally had good and smooth relationships with their main and regular DOTS service providers. Besides their warm greetings and hospitality, the providers’ attentive listening, enthusiastic understanding, polite answering as well as curious follow-up of their health status were among the indicators of smooth interaction and relationship commonly mentioned by participants of the study. However, few TB patients who had the experience of taking their medications on weekends (days off) by other professionals from other wards reported encountering unethical behaviors from the health care providers.

According to a previous local study, while supportive relation with health professionals contributed positively, lack of adequate communication was among barriers to treatment adherence [26, 27]. Another study in Addis Ababa also showed that DOTS was provided with limited patient-centered TB care [28]. Similarly, confirming patients’ relationship with treatment providers appeared to influence adherence, researches carried out in other parts of the world revealed that poor follow-up by providers in Indonesia and India [29, 30] and maltreatment by providers in India, Pakistan and Burkina Faso [17, 21, 31] resulted in non-adherence, whereas other studies set in Vietnam, Pakistan and Mexico noted the positive impact of increased provider-patient contact on adherence [10, 25, 32].

Adherence to treatment is facilitated by flexibility and patient choice regarding the number of professionals providing treatments, literatures suggested. Although more than half of the participants preferred treatment follow-ups provided by a single and the same professional, most of them reported receiving DOTS service by two or more health care providers. Such preferences for one and the same service provider throughout the treatment period were based on participants’ reasons that a new health professional might be unaware of previous problems, be uneasy to discuss and familiarize with, not refer to previously recorded medical record, and have miscommunication with existing service providers.

The participants generally evaluated the DOTS services they received as very good, and commonly mentioned health care providers’ good and respectful relationship with patients, free of charge provision of treatments, nearby location of centers, and generally instant provision of services as reasons behind their positive evaluation. Areas for future improvement suggested by patients included the needs to monitor and take corrective measures on health care providers working in other departments regarding their behavior and way of treating TB patients, avail distal water, refer difficult cases, increase number of professionals at different rooms to reduce the workload and patients’ queue, and to provide TB treatment service to a given patient by one and the same professional throughout the treatment period.

Medication side effect was the therapeutic theme identified and analyzed in this study. Most of the participants experienced side effects but also reported that the side effects disappeared after their adaptation to the medication, and only few told they did not experience any. Although a study mentioned experiencing side effects as barrier to adherence [26] and some other studies conducted in Asia noted that patients who felt worse than before treatment might be more likely to interrupt treatment [17, 21], no participant in the present research reported missing medication or having the intension to discontinue treatment as a result of medication side effects.

With regard to socioeconomic issues, while food accessibility and consumption represented economic influence on TB treatment adherence, social support and discrimination were the social factors considered in the study. Unlike the adherence barriers of lack of food and economic constraint reported in another study [26], the present study indicated that almost all the patients, except one, were able to get food whenever they needed to. Congruently, almost all participants, except one, reported the medication increased their appetite for food. In accordance with results demonstrating the presence of support from families positively influenced treatment adherence [26], almost all study participants reported having the required support from their respective family members. In line with reports stating family support, including financial assistance, collecting medication, and emotional support, appeared to be a strong influence on patient adherence to treatment [10, 24, 25, 30], patients interviewed in this study commonly mentioned getting necessary food items like milk and egg through their family members’ or colleagues/friends’ help in kind or finance as well as in preparation, reminders of medication time, and emotional support.

Finally, although most participants experienced no discrimination, few reported experiencing discriminations from family members due to the misinformation disseminated by reception and medical record room workers and from some community members due to lack of awareness and non-scientific personalistic understanding of TB causation. In comparison, a study reported that in some cases, patients on treatment became demoralized and non-adherent as social support weakened [21].

## Conclusions

The patients’ experiences and perceptions on associates of TB treatment adherence, based on an in-depth interview of ten TB patients receiving DOTS services in two public health centers in Addis Ababa, indicated that most patients adhered to their medications, but few experienced missing the exact time and doses of medications mainly due to forgetfulness. In addition to their understanding that TB is curable disease, participants knew the duration of treatment and side effects of medication as well as the negative consequences of discontinuing and effect of substance use on the therapeutic course.

Adding to factors that influenced adherence to DOTS positively, most patients were also well aware of the transmission routes and prevention mechanisms of TB. Nevertheless, most informants had misconceptions on TB causation. While perceived risks of non-adherence to medication was one of participants’ personal factor to adhere to the DOTS service, perceived wellness was the reason behind the intention they have had to discontinue their TB treatments.

The participants generally had positive evaluation of the patient-provider relationship and the health care obtained from DOTS service, albeit unethical behaviors of providers from other departments and reception rooms encountered by few TB patients. Even though more than half of study participants preferred treatment follow-ups provided by a single and the same professional, most of them received DOTS service by two or more health care providers. Physical accessibility of health facilities and the waiting time therein posed no difficulties in pursuing DOTS service follow-ups of all interviewed TB patients. In addition, although most interviewed TB patients experienced medication side effects that eventually disappeared after the intensive treatment phase, no participant reported missing or having the intension to discontinue medication due to this therapeutic factor.

Nearly all informants were able to get necessary food items and had increased appetite for food after commencing DOTS. Most also acquired required social supports and experienced no discrimination for being TB patient. However, some were discriminated by their family due to the overstated misinformation disseminated by supportive service providers to patients’ attendants and by some members of their community because of non-scientific understanding of TB causation.

The two health centers in which this qualitative study was conducted need to train and monitor occasional DOTS service providers from other departments regarding their behavior and way of treating TB patients, regularly avail distal water for injection, refer complicated cases, increase number of TB treatments rooms and regular DOTS service provider professionals, and to provide TB treatment service to a given patient by one and the same professional throughout the treatment period.

Findings of the study additionally implied the necessity of undertaking awareness creation activities, particularly regarding TB causation, targeted towards addressing both patients and members of their community. Moreover, social support, especially in reminding patients the time of medication, need to be improved as forgetfulness was a factor behind non-adherence.

## Abbreviations

DOTS: Directly observed treatment, short course
EPTB: Extra pulmonary tuberculosis
MDGs: Millennium development goals
MDR TB: Multi drug resistant tuberculosis
PTB: Pulmonary tuberculosis
TB: Tuberculosis
WHO: World Health Organization
